# Supplementary material for: A Highly Potent, Orally Bioavailable Pyrazole-Derived Cannabinoid CB2 Receptor- Selective Full Agonist for In Vivo Studies
Source: ACS Pharmacol Transl Sci. 2024 Jul 9;7(8):2424–38. doi: 10.1021/acsptsci.4c00269 (PMC11320734; doi:10.1021/acsptsci.4c00269)
Supplement: Supplementary file 1 — pt4c00269_si_001.pdf [file pt4c00269_si_001.pdf]

Supporting information

A highly potent, orally bioavailable pyrazole-derived cannabinoid CB2 receptor-selective full agonist for *in vivo* studies

Andrea Chicca[1], Daniel Bátora[1,2], Christoph Ullmer[3], Antonello Caruso[3], Sabine Grüner[3], Jürgen Fingerle[3], Thomas Hartung[3], Roland Degen[3], Matthias Müller[3], Uwe Grether[3], Pal Pacher[4*], Jürg Gertsch[1*]

[1] *Institute of Biochemistry and Molecular Medicine, University of Bern, Bern 3012, Switzerland*

[2] *Graduate School for Cellular and Biomedical Sciences, University of Bern, Bern, Switzerland*

[3] *Pharmaceutical Sciences, Roche Innovation Center Basel, Roche Pharma Research and Early Development, Basel 4070, Switzerland*

[4] *Laboratory of Cardiovascular Physiology and Tissue Injury (P.P.), National Institute on Alcohol Abuse and Alcoholism, National Institutes of Health (NIH), Bethesda, MD.*

*Correspondence:

Jürg Gertsch, PhD
Institute of Biochemistry and Molecular Medicine, University
of Bern
Email: juerg.gertsch@unibe.ch

Pal Pacher MD, PhD, FAHA, FACC
Laboratory of Cardiovascular Physiology and Tissue Injury,
NIAAA/NIH, Bethesda, US
Email: pacher@mail.nih.gov

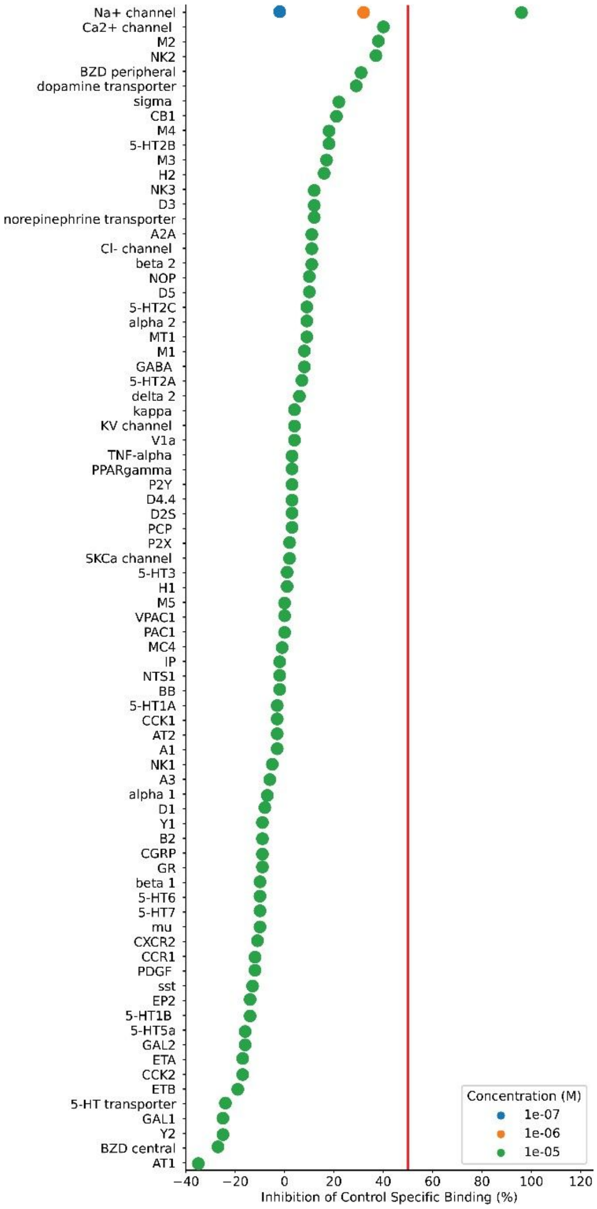

Figure S1: CEREP screening for off-targets

The plot depicts the binding affinity of 0.1-10 µM of **RNB-61**, expressed as the percentage of inhibition of control specific binding on 80 receptors. The threshold for follow-up was set to 50% at 10 µM concentration. Only the Na+ channel showed strong RNB-61 binding (96%) at 10 M, therefore, a follow-up of 1 µM and 0.1 µM was conducted, which showed 32% and 2% inhibition, respectively.

	RNB-61 (K_i, IC_{50})
FAAH	>10000
MAGL	>10000
ABHD6	>10000
ABHD12	>10000
EMT	>10000
COX-2	>10000
TRPV1	>50000
TRPA1	>50000
GPR55	>50000
PPARg	>10000

Table S1: RNB-61 shows no binding interaction and functional inhibition on endocannabinoid related target proteins.

Abbreviations: FAAH, Fatty-acid amide hydrolase 1; MAGL, Monoacylglycerol lipase; ABHD, alpha/beta-Hydrolase domain containing; EMT, Endocannabinoid membrane transporter; COX-2, Cyclooxygenase-2; TRPV1, Transient receptor potential vanilloid 1; TRPA1, Transient receptor potential cation channel, subfamily A, member 1; GPR55, G protein-coupled receptor 55, PPARG, Peroxisome Proliferator Activated Receptor Gamma

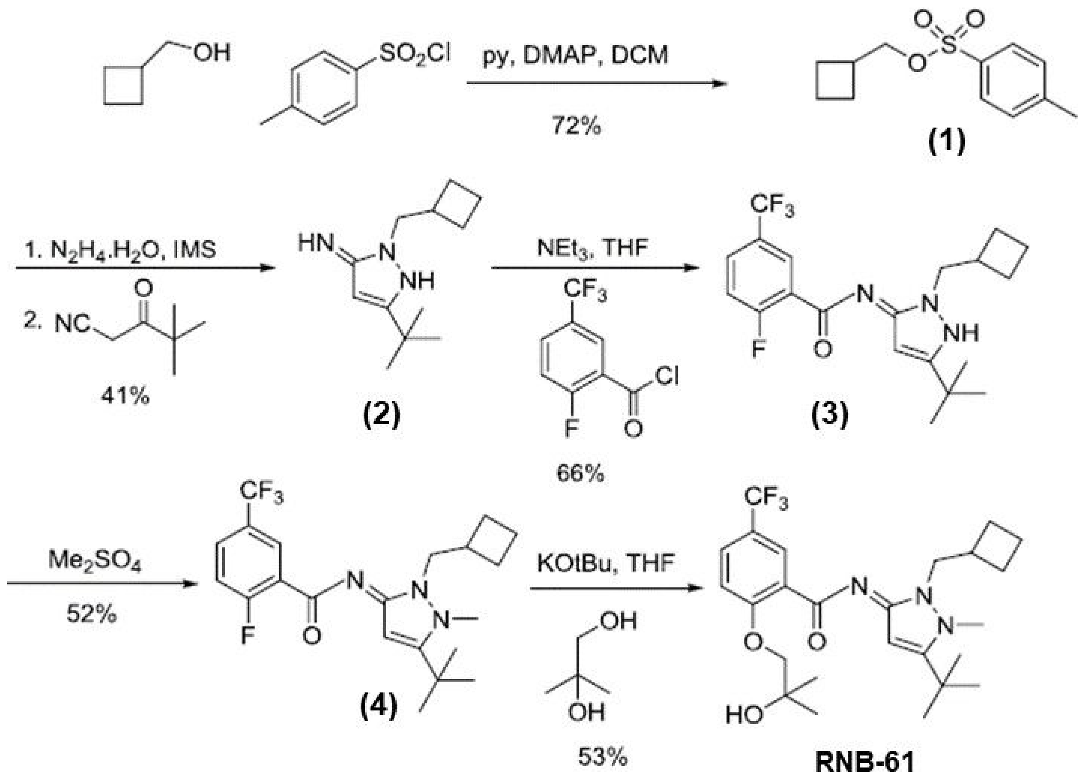

Scheme S1: Synthesis of RNB-61

Detailed synthesis procedures

*Preparation of N-(5-tert-Butyl-2-(cyclobutylmethyl)-1-methyl-1H-pyrazol-3(2H)- ylidene)-2-(2-hydroxy-2-methylpropoxy)-5-(trifluoromethyl)benzamide (**RNB-61**)*

*Preparation of 1-(2-Cyclobutylethylsulphonyl)-4-methylbenzene (**1**):* To a solution of cyclobutylmethanol (150 g, 1.74 mol) and pyridine (600 mL) in dichloromethane (1.5 l) was added p-toluenesulphonyl chloride (332 g, 1.74 mol). The solution was allowed to stand overnight and was then treated with 5% hydrochloric acid (180 mL). The organic layer was separated, dried and concentrated. Dry flash chromatography (ethyl acetate → heptane) gave **(1)** as a colourless oil (303 g, 72%).

Preparation of 5-tert-Butyl-2-(cyclobutylmethyl)-2,3-dihydro-1H-pyrazol-3-amine (2): A mixture of **(1)** (303 g, 1.26 mol), hydrazine hydrate (93 g, 1.9 mol) and IMS (1.5 L) was heated at reflux for 8 h, cooled to room temperature, treated with trimethylacetylacetonitrile (237 g, 1.9 mol) and heated at reflux for a further 5 h. On cooling the solution was concentrated and treated with dichloromethane (3 L). The resultant solution was stirred with saturated aqueous sodium bicarbonate (1.5 L). The organic layer was separated, dried and concentrated to an oil. Dry flash chromatography (ethyl acetate → heptane) gave a solid, which was recrystallised from hexane to give **(2)** as an off-white solid (108 g, 41%).

Preparation of N-(5-tert-Butyl-2-(cyclobutylmethyl)-1H-pyrazol-3(2H)-ylidene)-2- fluoro-5-(trifluoromethyl)benzamide (3): To a solution of **(2)** (108 g, 0.52 mol) and triethylamine (210g, 2.1 mol) in THF (3 L) was added dropwise over 0.5 h to a solution of 2-fluoro-5-trifluoromethylbenzoyl chloride (118 g, 0.52 mol) in THF (500 mL). On stirring for a further 0.5 h, water (4 L) was added and the product was extracted into dichloromethane (2 × 1500 mL), washed with water (2 × 1 L), dried and concentrated to an oil. Dry flash chromatography (ethyl acetate → heptane) gave **(3)** as a white solid (130 g, 66%).

Preparation of N-(5-tert-Butyl-2-(cyclobutylmethyl)-1-methylpyrazol-3(2H)-ylidene)-2-fluoro-5-(trifluoromethyl)benzamide (4): **(3)** (130 g, 0.33 mol) and dimethyl sulphate (1.3 L) were stirred at 100°C for 24 h, cooled and poured into 5% aqueous ammonia (7 L). The product was extracted into ethyl acetate (3 × 1 L) and the organic fraction was separated, washed with water (2 L) then brine (500 mL), dried and concentrated. Dry flash chromatography (ethyl acetate → heptane) gave **(4)** as an oil (71 g, 52%) that solidified upon standing.

*Preparation of N-(5-tert-Butyl-2-(cyclobutylmethyl)-1-methyl-1H-pyrazol-3(2H)- ylidene)-2-(2-hydroxy-2-methylpropoxy)-5-(trifluoromethyl)benzamide (**RNB-61**):* To a solution of 2-methylpropane-1,2-diol (34 g, 0.38 mol) in THF (2 L) was added potassium tert-butoxide (39

g, 0.35 mol). On stirring for 0.5 h a solution of **(4)** (71 g, 0.173 mol) in THF (500 mL) was added. The mixture was stirred overnight then poured into water (2 L). The product was extracted into ethyl acetate (2 × 1500 mL), washed with water (2 x 2 L), dried and concentrated to an oil. Recrystallisation from toluene gave **RNB-61** as a white solid (45 g, 53%), mp 191- 192°C (eff). The reaction of the amide with dimethyl sulfate in the presence of potassium carbonate led selectively to amide nitrogen methylation yielding the inactive regioisomer of **RNB-61**.
